# The Mesenteric Glands in Relation to Tuberculosis

**Published:** 1905-09-16

**Authors:** 


### THE MESENTERIC GLANDS IN RELATION TO TUBERCULOSIS.

Dr. Randle Rosenberger[1] has conducted an important investigation into the frequency with which the mesenteric lymphatic glands are infected by the tubercle bacillus. The study is based upon an examination of 70 bodies. These he has divided into two classes according to the presence or absence in the individuals of gross, naked-eye evidence of tuberculous lesions anywhere in the body. Of the total, 49 presented in one part or other gross evidence of the disease. For descriptive purposes the mesenteric glands are reckoned in the following three categories: 1. Normal, that is, appearing normal to the eye. 2. Enlarged, but without gross evidence of tuberculosis. 3. Tuberculous, that is, obviously so.

Of the 49 tuberculous cases seven exhibited normal mesenteric glands; in 22 cases the glands were enlarged, and in 20 tuberculous. But further investigation revealed that a mere judgment from the gross characteristics was most fallacious, for upon making "spread" preparations from the glands it was found that 36 per cent. of the glands, which upon such treatment were found to contain tubercle bacilli, yet gave no macroscopic evidence of their infection. The glands from 24 of these tuberculous cases which yielded no tubercle bacilli in "spread" preparations, were then examined by the inoculation of guinea-pigs with an emulsion of the tissue. Ignoring nine of these experiments, in which the early deaths of the guinea-pigs precluded a definite conclusion, all but one showed that the glands employed were the site of living tubercle bacilli. The only animal which yielded a negative result was one injected with a gland-emulsion from a patient whose sole evidence of past tuberculosis consisted of a scar at the apex of one lung. Thus, again excluding the indeterminate cases above-mentioned, 39 out of 40, or over 97 per cent., of the glands employed were virulent to guinea-pigs, although there was in many of them no gross evidence of the infection.

In the non-tuberculous group examination of the mesenteric glands for tubercle bacilli by means of "spread" preparations was in all cases followed by a negative result. None the less injection of emulsions from these glands resulted in the production of tuberculosis in guinea-pigs in 40 per cent. of the cases. Similarly enlarged axillary lymphatic glands from a case of chronic pulmonary tuberculosis were found capable of producing the disease upon injection into a susceptible animal, as were the apparently normal inguinal glands of a patient dying from miliary tuberculosis. As regards the virulence of the viscera it appeared that emulsions of liver, spleen, and kidney, from a case of chronic pulmonary tuberculosis could produce tuberculosis in guinea-pigs, while similar emulsions taken from cases in the non-tuberculous series failed in every case to reproduce the disease.

The author's main conclusions are as follows:—

1. In all cases of active tuberculosis, and in almost all cases of inactive tuberculosis, the mesenteric glands are tuberculously infective.

2. The mesenteric glands in these cases may or may not show gross evidence of tuberculosis, and the same statement applies to "spread" preparations designed to demonstrate the bacillus microscopically.

3. The mesenteric glands in a certain percentage of cases showing no tuberculous lesion in any part of the body, are tuberculously infective. In the present study the percentage was about 40.

4. The tuberculous infectivity of the mesenteric glands is probably shared by the other groups of lymph-nodes throughout the body.

The bearing of these investigations upon the development of abdominal tuberculosis deserves a few words of comment. The presence of living tubercle bacilli in the mesenteric glands of a large percentage of those in whom no lesions of tuberculosis could be found in any part of the body, proves almost to demonstration that primary infection by the intestinal route is a commonplace rather than the rarity which Koch would have us believe it to be, whether or not such glandular infection postulates a previous lesion of the mucous surface is an academic matter; for the transition between catarrhal desquamation of epithelium, or even the normal desquamation of the healthy intestine, and a desquamation deserving to be called ulceration, is very gradual, and a temporary breach of surface may well admit tubercle bacilli to the lymphatics and yet leave no trace of its previous existence. The source of the infection in cases of what appears to be primary tuberculosis of the peritoneum, as of the pleura, is, and will probably remain to a large extent, a matter of inference rather than of demonstration. These infections are generally miliary in character, and quite commonly limited absolutely to the serous membrane; that is to say the inner tissues of the viscera may show no sign of the disease. Now the alternative possibilities in such a case are these three. The infection may come by the blood-stream, or by the lymphatics, or by a surface contamination of the serous membrane owing to the discharge of tuberculous material into the serous cavity. The absence of tissue-lesions in the viscera seems to eliminate the possibility of infection by direct extension of the tuberculous process. Now a miliary infection of the peritoneum, involving, as it does in many cases, a large, if not the whole, surface of the membrane, indicates that a large number of bacilli have been simultaneously disseminated over a large area. Such a comprehensive dissemination can only be effected by two means; either the blood-stream is charged with bacilli, or the rupture of a caseous focus into the serous cavity has caused a generalised surface infection. But if the blood-stream of the abdominal cavity be charged with bacilli to such an extent as to produce thousands of isolated foci on the peritoneal surface, how comes it that the organs served with blood from the same systemic supply very often show no evidence of infection? Unless some fallacy has escaped us the inference is emphatic that what is called primary, miliary, tuberculous peritonitis depends upon the rupture into the peritoneal sac of a caseous focus charged with living bacilli.

What is really required for the settlement of this question is that surgeons called upon to perform laparotomy for the ascites commonly associated with these cases of miliary tuberculous peritonitis should undertake a systematic search for evidence of mesenteric glandular caseation. If macroscopic evidence of such caseation were forthcoming in even a moderate proportion of the cases so examined, the inference of its universality, and of its real responsibility for the peritoneal lesions, would be more than justifiable. We know from the researches of Dr. Rosenberger that the mesenteric glands are quite commonly inhabited by living and virulent tubercle bacilli even when they preserve the appearance of normal health; we have seen also the difficulties in the way of accepting a circulatory origin for the multitudinous focal infections of this disease, and the assumption is legitimate that, if in a certain number caseation of the mesenteric glands is demonstrable by the unaided eye, in the remainder such caseation is present though latent. When it is remembered that a small caseous focus may contain a vast number of virulent tubercle bacilli, it is not hard to credit that the rupture of such a focus into the peritoneal cavity, while it escapes notice, is none the less a possible source of sufficient bacilli to produce the high numerical count of isolated lesions common to the disease.

[1] Amer. Jour. of Med. Sciences, July 1905.

